# Comparative analyses reveal discrepancies among results of commonly used methods for *Anopheles gambiae*molecular form identification

**DOI:** 10.1186/1475-2875-10-215

**Published:** 2011-08-02

**Authors:** Federica Santolamazza, Beniamino Caputo, Maria Calzetta, José L Vicente, Emiliano Mancini, Vincenzo Petrarca, João Pinto, Alessandra della Torre

**Affiliations:** 1 Istituto Pasteur-Fondazione Cenci-Bolognetti, Dipartimento di Sanità Pubblica e Malattie Infettive, Università SAPIENZA, Piazzale Aldo Moro 5, 00185, Rome, Italy; 2 UEI Parasitologia Médica, Centro de Malária e outras Doenças Tropicais, Instituto de Higiene e Medicina Tropical, Universidade Nova de Lisboa, Rua da Junqueira, 100, 1349-008 Lisbon, Portugal; 3 Istituto Pasteur-Fondazione Cenci-Bolognetti, Dipartimento di Biologia e Biotecnologie, Università SAPIENZA, Piazzale Aldo Moro 5, 00185, Rome, Italy

## Abstract

**Background:**

*Anopheles gambiae *M and S molecular forms, the major malaria vectors in the Afro-tropical region, are ongoing a process of ecological diversification and adaptive lineage splitting, which is affecting malaria transmission and vector control strategies in West Africa. These two incipient species are defined on the basis of single nucleotide differences in the IGS and ITS regions of multicopy rDNA located on the X-chromosome. A number of PCR and PCR-RFLP approaches based on form-specific SNPs in the IGS region are used for M and S identification. Moreover, a PCR-method to detect the M-specific insertion of a short interspersed transposable element (*SINE200*) has recently been introduced as an alternative identification approach. However, a large-scale comparative analysis of four widely used PCR or PCR-RFLP genotyping methods for M and S identification was never carried out to evaluate whether they could be used interchangeably, as commonly assumed.

**Results:**

The genotyping of more than 400 *A. gambiae *specimens from nine African countries, and the sequencing of the IGS-amplicon of 115 of them, highlighted discrepancies among results obtained by the different approaches due to different kinds of biases, which may result in an overestimation of MS putative hybrids, as follows: i) incorrect match of M and S specific primers used in the allele specific-PCR approach; ii) presence of polymorphisms in the recognition sequence of restriction enzymes used in the PCR-RFLP approaches; iii) incomplete cleavage during the restriction reactions; iv) presence of different copy numbers of M and S-specific IGS-arrays in single individuals in areas of secondary contact between the two forms.

**Conclusions:**

The results reveal that the PCR and PCR-RFLP approaches most commonly utilized to identify *A. gambiae *M and S forms are not fully interchangeable as usually assumed, and highlight limits of the actual definition of the two molecular forms, which might not fully correspond to the two *A. gambiae *incipient species in their entire geographical range. These limits are discussed and operational suggestions on the choice of the most convenient method for large-scale M- and S-form identification are provided, also taking into consideration technical aspects related to the epidemiological characteristics of different study areas.

## Background

The mosquito vector species responsible for most *Plasmodium falciparum-*malaria transmission in sub-Saharan Africa, *Anopheles gambiae *sensu stricto (hereafter *A. gambiae*), is ongoing a process of ecological diversification and adaptive lineage splitting which is changing patterns of malaria transmission and affecting vector control strategies in West Africa [1-4]. Two morphologically indistinguishable incipient species (provisionally named M and S molecular forms) have been described within *A. gambiae*, based on form-specific single nucleotide polymorphisms (SNPs) on the intergenic spacer (IGS) and internal transcribed spacer (ITS) regions of multicopy ribosomal DNA (rDNA) located on the X-chromosome [5,6]. S-form is distributed across sub-Saharan Africa and breeds mostly in association with rain-dependent pools and temporary puddles. M-form distribution overlaps with that of S-form in West and Central Africa, but the former form is apparently absent east of the Great Rift Valley; it is able to exploit relatively more permanent breeding sites, often closely associated with human activities, such those created by irrigation, rice cultivation and urbanization [2,3,7,8]. This adaptation allows the M-form to breed throughout the year, thus causing a shift from seasonal to year-round malaria transmission. Importantly, genetic traits conferring resistance to insecticides commonly used against these vectors are differently distributed between the two forms [9,10].

**Figure 1:**
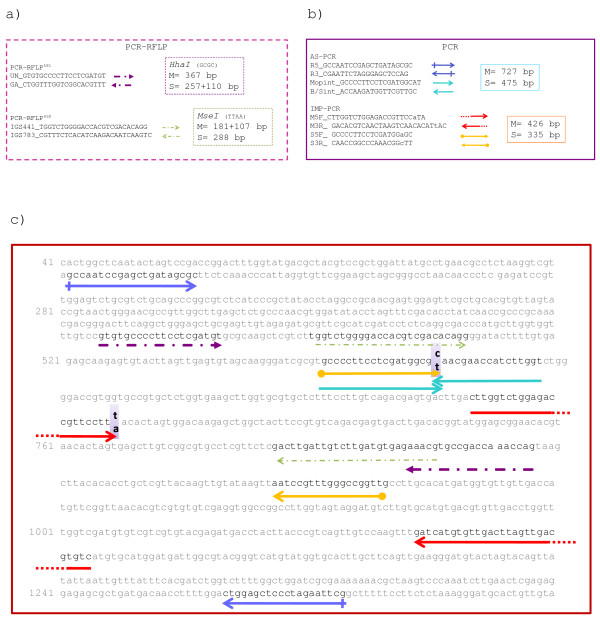
**Sequence and alignment of M and S *Anopheles gambiae *molecular form specific diagnostic primers**. a) Primer sequences, restriction enzymes and M and S *Anopheles gambiae *molecular form specific products from PCR-RFLP^581^[16] and PCR-RFLP^690 ^[16,17]; b) primer sequences and molecular form-specific products as in AS-PCR [13] and IMP-PCR [14]; c) location of primer pairs and restriction sites utilized in AS-PCR and PCR-RFLPs are reported on the 28S (from 41 to 400) IGS sequence (from 401 to 1321) (AF470093-AF470116; [29]).

Genetic divergence between M and S forms has been recently shown to be widespread across the genome [11,12]. However, the most widely used methods for M and S specimen identification are based on genotyping procedures for the form-specific SNPs in the IGS rDNA region on the centromere of the X-chromosome. These are performed either by PCR using form-specific primers [13,14] or PCR-RFLP [15-17] (Figure 1). More recently, a PCR-method to detect the M-specific insertion of a *SINE200 *(short interspersed transposable element), mapping about 1 Mb apart from IGS SNPs in the chromosome-X centromeric region, was also developed [18]. Results from large scale identification of *A. gambiae *field specimens by means of any of these approaches highlighted the virtual absence of hybrid M/S patterns, thus contributing substantial evidence of M and S reproductive isolation in nature. However, high frequencies of M/S IGS-patterns have been recently reported from The Gambia [19] and Guinea Bissau [20]. A preliminary comparison of different identification approaches in samples from these westernmost geographical areas highlighted inconsistencies in the results and the occurrence of possible biases due to the routine practice of identifying M and S mosquitoes based on a single assay [21].

The results of a large-scale comparative analysis of four widely used PCR or PCR-RFLP genotyping methods for M and S identification provide operational recommendations to medical entomologists dealing with M and S identification in the Afrotropical region.

## Methods

### Samples, genotyping and sequencing

**Figure 2:**
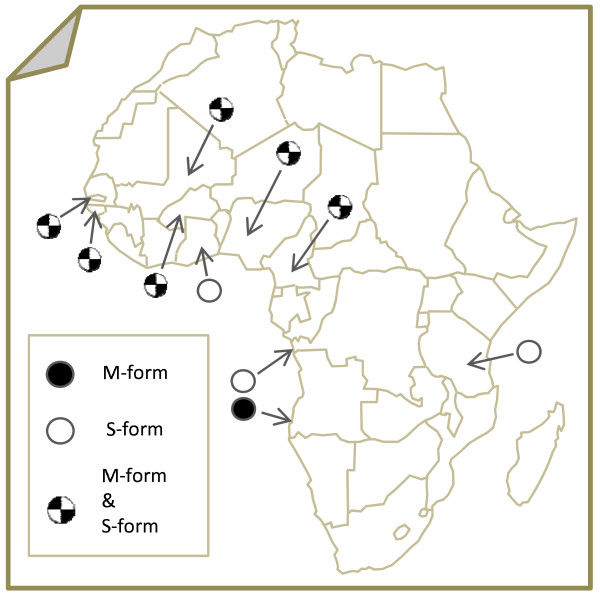
**Location of collection sites**. Black and white pies indicate the exclusive presence of either M or S *Anopheles gambiae *molecular forms, respectively. Black/white pies indicate sites where both molecular forms were sampled.

Table 1 lists the indoor-resting female samples processed in this study, the dates of collections and the numbers of specimens genotyped. Figure 2 shows the location of the sampling sites. The specimens from Guinea Bissau and The Gambia [21] were selected based on inconsistent results from IGS [16] and *SINE200 *insertion [18] genotyping.

**Table 1:** Collection sites of analysed *Anopheles gambiae *s.s. indoor-resting female samples, and references to published papers.

Countries	Collection sites	Longitude	Latitude	Year of collection	N	Reference
**Tanzania**	Nyakariro, Kwagole	05°05', 02°30'S	39°08'-33°27'E	1997-98	28	[7]
**Angola**	Cabinda, Luanda	05°32', 08°50'S	12°11', 13°14'E	2003	64	[30]
**Cameroon**	Mangoum, Kribi	05°31'-02°56'N	9°54'-10°37'E	2005-06	47	[18]
**Nigeria**	Kobape, Olugbo	07°00' -07°20'N	03°00' -03°30'E	2001	27	[31]
**Burkina Faso**	Bobo Dioulasso	11°02'N	04°13'W	2001	58	[7]
**Mali**	Banambani	12°48'N	08°03'W	1996	39	[7]
**Ghana**	Accra area	05°38'N	00°15'W	2002	45	[18]
**Guinea Bissau**	Antula	11°5'N	15°30'W	1995	32	[20]
				2007	35	[20]
**The Gambia**	Kartong, Sare Samba Sowe	13°05'-13°34' N	16°45'-15°54'W	2006	49	[19]

N = number of specimens/sample.

DNA was extracted from either legs or other parts of the carcasses not including the abdomen, to avoid possible biases associated to the risk of contamination with DNA from sperm harboured in spermathecae.

*Anopheles gambiae *samples were first identified based on results from PCR-RFLP approach (from now on PCR-RFLP^581^) recognizing a T/C SNP (T = M-form; C = S-form) at position 581 of IGS rDNA region (from now on IGS^581^[16]). Subsequently, the following genotyping approaches were applied (Figure 1 shows the position of primers and restriction sites on the IGS amplicon sequence): i) the PCR-RFLP approach (from now on PCR-RFLP^690^) recognizing a A/T SNP (A = M-form; T = S-form) at position 690 of IGS rDNA region (from now on IGS^690 ^[17]); ii) the PCR approach using allele-specific primers designed to detect the IGS^581 ^SNP (from now on AS-PCR [13]); iii) the PCR approach based on the specific and irreversible single-locus insertion of a *SINE200 *transposable element in the X-chromosome centromeric region (from now on SINE-PCR [18]), about 1 Mb from the IGS rDNA region including the IGS form-specific SNPs. A sub-sample of specimens from Angola, Burkina Faso, The Gambia and Guinea Bissau were also identified by a PCR approach utilizing Intentional Mismatch Primers containing single base mismatches at the third nucleotide from their 3' end (from now on IMP-PCR[14]).

An IGS fragment of 367 bp (from now on "IGS-amplicon") was amplified using UN and GA primers by Fanello *et al *[16] (Figure 1) from selected specimens and sequenced using ABI Big Dye Terminator v.2 chemistry and an ABI Prism 3700 DNA Analyser. Chromatograms were inspected for double peaks by eye. PCR and sequence analyses were carried out in Rome and/or Lisbon. Selected samples were analysed in both laboratories for results validation.

### Statistical analyses

QSVanalyzer software - which allows the extraction of quantitative sequence variant (QSV) information from sequence electropherograms - was applied to estimate the relative proportions of the double peaks (*i.e*., **c**opy **n**umber **p**roportions: CNP)[22]) observed in electropherograms of IGS amplicon at positions 581 [16] (hereafter CNP^581^) and 690 [17] (hereafter CNP^690^) in sequences of the IGS locus from single *A. gambiae *specimens. The programme analyses each trace and adjusts it in relation to the peak heights of upstream/downstream nucleotides, allowing rapid batchwise analysis of DNA sequence traces for estimation of the relative proportions of two QSVs at a given site. Kruskal-Wallis and multiple comparison tests were carried out by STATISTICA 6.1 (StatSoft, Inc. 2003).

## Results

Four-hundred-twenty-four *A. gambiae *specimens from nine African countries by PCR-RFLP^581^, PCR-RFLP^690^, AS-PCR and SINE-PCR were genotyped (Table 1). Most (97%) of the specimens were consistently identified by all approaches in samples from Tanzania to Ghana (N = 250), while the percentage of consistent identifications was lower (46%) in samples from Guinea Bissau and The Gambia - which were selected based on previous inconsistent results from PCR-RFLP^581 ^and SINE-PCR [21] - and in samples from Burkina Faso (41%). Inconsistent identifications were confirmed at least twice by PCR and PCR-RFLP genotyping carried out in different laboratories. The IGS-amplicon was sequenced in 115 specimens (Angola N = 5; Cameroon N = 1; Nigeria N = 1; Burkina Faso N = 16; Mali N = 1; Guinea Bissau N = 62; The Gambia N = 29). The latter samples plus additional 110 specimens (Tanzania N = 11; Angola N = 21; Cameroon N = 9; Nigeria N = 11; Burkina Faso N = 20; Mali N = 12; Ghana N = 18; Guinea Bissau N = 1; The Gambia N = 7), were also genotyped by IMP-PCR.

The results were as follows:

**Tanzania**. All specimens were consistently identified as S-form by the four approaches utilized. Eleven identifications were confirmed also by IMP-PCR.

**Angola**. All individuals from Cabinda (N = 32) were identified as S-form by the four approaches. Twenty-seven out of 32 individuals from Luanda were identified as M-form by all approaches, while five of them showed a MS^690 ^heterozygous pattern. Sequence analysis of these specimens revealed the presence of an A/C polymorphism at position 690 (instead of the expected A/T polymorphism, corresponding to the *MseI *restriction site), which does not allow the form-specific cleavage of the PCR-amplified band. Ten M- (including the above 5) and 11 S-identifications were confirmed also by IMP-PCR.

**Cameroon**. Twenty individuals were consistently identified as M-form and 26 as S-form. One single specimen showed a MS^690 ^pattern, but a M-form pattern by the other approaches. IMP-PCR and IGS-sequencing confirmed the MM genotype.

**Nigeria**. Nine individuals were consistently identified as M-form and 17 as S-form. One single specimen showed a S-pattern by both PCR-RFLPs and MS by AS-PCR. IMP-PCR and IGS-sequencing confirmed the SS genotype.

**Burkina Faso**. Eleven individuals were consistently identified as M-form and 13 as S-form; 32 specimens (MM^581^-MM^690 ^= 17 and SS^581^-SS^690 ^= 15) showed a MS-pattern only by AS-PCR. The PCR-RFLP genotypes were confirmed either by IMP-PCR and/or by sequencing of the IGS-amplicon, which did not reveal any MS-heterozygous pattern. Two MM^581^-MS^690 ^specimens were found, one showing MS-AS-PCR, the other one showing M-AS-PCR pattern. Both were genotyped as MM by IMP-PCR and IGS-sequencing.

**Mali**. Three individuals were consistently identified as M-form and 35 as S-form. One single specimen showed a MS-pattern by AS-PCR and a M-form pattern by the other approaches: both IMP-PCR and IGS-sequencing confirmed the MM genotype.

**Ghana**. All specimens were consistently identified as S-form by the four approaches utilized.

**Guinea Bissau**. Results from PCR-RFLP^581^, PCR-RFLP^690^, AS-PCR and sequencing are shown in Table 2: 61% (41/67) of the specimens showed consistent genotypes with the three approaches (i.e. 5 M, 29 MS, 7 S; Table 2, lines 1, 5, 10). PCR-RFLP^581^and PCR-RFLP^690 ^patterns were congruent in 73% (49/67, lines 1, 2, 5,7,10) of the specimens: the remaining were either MM^581^-MS^690 ^(10%; lines 3 and 4) or MS^581^-SS^690 ^(16%, lines 6, 8 and 9). No opposite MM^581^-SS^690 ^nor SS^581^-MM^690 ^identifications were observed. An AS-PCR heterozygous MS pattern was obtained from: i) all specimens genotyped as heterozygotes by at least one PCR-RFLP, with the exception of four MS^581^-SS^690 ^specimens showing a SS AS-PCR pattern (line 9); ii) 6 out of 11 MM^581^-MM^690 ^homozygotes (line 2); and iii) 2 out of 9 SS^581^-SS^690 ^(line 7). A match between the results of SINE-PCR and IGS genotypes (as defined by either consistent results of the different genotyping approaches and/or by direct sequencing of the IGS-amplicon, see below) was observed in 69% of the specimens (46/67: 10 MM, 25 MS and 11 SS). The mismatches were either due to SS (16/21) and MM (1/21) SINE-homozygotes with a heterozygous MS^IGS ^genotype, and to MS SINE-heterozygotes with a homozygous MM^IGS ^(1/21) or SS^IGS ^(3/21) genotype.

**Table 2:** Results of identification of *Anopheles gambiae *s.s. indoor-resting female samples by different genotyping approaches

Samples		PCR-RFLPs				Sequencing	
		**IGS**^**581**^	**IGS**^**690**^	AS-PCR	N (n)	**IGS**^**581**^	**IGS**^**690**^
	1	MM	MM	MM	5 (4)	T	A
	2	MM	MM	MS	6 (3)	T	A
	3	MM	MS	MS	1	T	A
	4	MM	MS	MS	6	T/C	A/T
	5	MS	MS	MS	29 (28)	T/C	A/T
**GUINEA BISSAU**	6	MS	SS	MS	6	T/C	A/T
	7	SS	SS	MS	2	T/C	A/T
	8	MS	SS	MS	1	C	T
	9	MS	SS	SS	4	C	T
	10	SS	SS	SS	7	C	T

	11	MM	MM	MM	9 (4)	T	A
	12	MM	MM	MS	7 (4)	T	A
	13	MM	MS	MM	1	T	A
	14	MM	MS	MS	1	T	A
**THE GAMBIA**	15	MM	MS	MS	2	T/C	A/T
	16	MS	MS	MS	16 (8)	T/C	A/T
	17	MS	SS	MS	1	T/C	A/T
	18	MS	SS	MS	2	C	T
	19	SS	SS	MS	1	C	T
	20	SS	SS	SS	9 (5)	C	T

PCR-RFLP^581^[16], PCR-RFLP^690 ^[17] and AS-PCR [13] genotyping and sequencing of IGS amplicon. IGS^581^: M-form = T, S-form = C; IGS^690^: M-form = A, S-form = T. N = numbers of specimens identified by PCR-RFLPs and AS-PCR. (n) = number of specimens sequenced, when these do not correspond to N.

**The Gambia**. Results from PCR-RFLP^581^, PCR-RFLP^690^, AS-PCR and sequencing are shown in Table 2: 69% (34/49) of the specimens analysed showed consistent genotypes with the three approaches (i.e. 9 M, 16 MS, 9 S; Table 2, lines 11, 16, 20). PCR-RFLP^581^and PCR-RFLP^690 ^patterns were congruent in 86% (42/49) of the specimens. The remaining were either MM^581^-MS^690 ^(8%; lines 13-15) or MS^581^-SS^690 ^(6%; line 17-18). No MM^581^-SS^690 ^nor SS^581^-MM^690 ^genotypes were observed. An AS-PCR heterozygous MS pattern was obtained for: i) all specimens genotyped as heterozygotes by at least one PCR-RFLP, with the exception of one MM^581^-MS^690 ^specimens showing a MM AS-PCR pattern (line 13); ii) 7 out of 16 MM^581^-MM^690 ^homozygotes (line 12) and 1 out of 10 SS^581^-SS^690 ^(line 19). A match between the results of SINE-PCR and IGS genotype (as defined from consistent results of the different genotyping approaches and/or by direct sequencing of the IGS-amplicon, see below) was observed in 72% of the specimens (N = 36: 16 MM, 9 MS and 11 SS). The mismatches were either due to SS (9/13) and MM (1/13) SINE-homozygotes with a heterozygous MS^IGS ^genotype or to MS SINE-heterozygotes with MM^IGS^(2/13) or SS^IGS^(1/13) genotypes.

**Figure 3:**
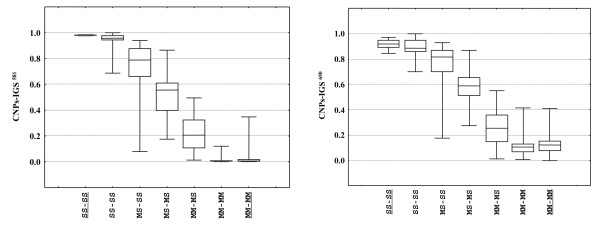
**Box-plots of CNP scores of IGS^581 ^SNP (a) and IGS^690 ^SNP (b) in *Anopheles gambiae *specimens**. Specimens are classified based on results from IGS^581^/IGS^690 ^PCR-RFLPs. The underlined SS/SS (N = 9) and MM/MM (N = 15) groups correspond to S-form and M-form specimens from Burkina Faso and Angola, while the not-underlined groups correspond to specimens from The Gambia and Guinea Bissau (SS-SS: N = 15; MS-SS: N = 14; MS-MS: N = 36; MM-MS: N = 11; MM-MM: N = 15).

The electrophoregrams of the overall sequenced sample were further scored by QSV analyser [22] to quantify the proportion of sequences containing C *versus *T (M-form = T; S-form = C) or A *versus *T (M-form = A; S-form = T), based on relative peak heights at position 581 (CNP^581^) and 690 (CNP^690^), respectively. As expected based on the proximity of the IGS^581 ^and IGS^690 ^SNPs, the CNP scores were strongly correlated (r = 0,97 p << 0.001). The median CNP scores of the two SNPs were significantly different among the 7 IGS-types classified based on both IGS^581 ^and IGS^690 ^PCR-RFLPs (IGS^581^: KW-H = 116, p << 0.001; IGS^690^: KW-H = 109, p << 0.001), with specimens identified as MM and SS by both PCR-RFLPs showing median CNP scores near 0.1 and 0.9, respectively (as expected if one allele is fixed) and specimens identified as MS by both PCR-RFLPs showing intermediate scores (Figure 3). The heterozygotes (MS^581^-MS^690^) were statistically different from the homozygotes (pairwise comparisons: p < 0.001; Additional file 1). Interestingly, specimens from Guinea Bissau and The Gambia, characterized by inconsistent PCR-RFLP patterns showed intermediate scores between those of M or S homozygotes (MM^581^-MM^690 ^or SS^581^-SS^690^) and MS heterozygotes (MS^581^-MS^690^), suggesting that these specimens are characterized by an unequal number of copies of M- and S-arrays (pairwise comparisons: p > 0.05; Additional file 1). CNPs scores of individuals subdivided based on SINE-genotypes revealed that some SINE-X^MM ^and SINE-X^SS ^individuals are characterized by an unequal number of copies of M- and S-arrays [21]. It is relevant to note that the interpretation of single versus double peaks at the two IGS diagnostic sites determined either by eye inspection of chromatograms or by CNP score (i.e. SNP^581^: homozygous T, CNP < 0.15; homozygous C, CNP > 0.85; SNP^690^: homozygous A, CNP < 0.18; homozygous T, CNP > 0.85) were consistent in 97% of the cases. IMP-PCR confirmed the results from sequencing, with the exception of 2 S-form individuals from The Gambia genotyped as MS by both IMP-PCR and AS-PCR.

Sequence analysis also showed that all samples were homozygotes (G) at position IGS^580^, where Favia *et al *[13] reported an addtional form-specific SNP (M-form = G; S-form = A). Moreover, alignment of IGS amplicon from sequenced individuals from Guinea Bissau (M-form: N = 31; S-form: N = 32), showed 4 IGS-polymorphic sites in addition to IGS^581 ^and IGS^690^: i) site IGS^485 ^was found heterozygous (C/T) in one M/S specimen; ii) site IGS^489 ^was found heterozygous (G/A) in 2 M and in 1 M/S specimens; iii) site IGS^491 ^was found homozygous (T) in one M specimen and heterozygous (T/C) in 2 M/S specimens. Finally, a A/G polymorphism at site IGS^612 ^was found in linkage with both IGS^581 ^and IGS^690 ^diagnostic sites, as already reported by Oliveira *et al *[20].

## Discussion

Since their description 10 years ago [5], *A. gambiae *M and S molecular forms have been the focus of extensive field studies aimed to evaluate their macro- and micro-geographic distribution and to analyse their population genetics, their relative role as malaria vectors and their resistance to insecticides used in malaria vector control campaigns. All these studies have exploited different approaches for M and S identification - based on either direct PCR-amplification of form-specific bands by allele-specific primers designed in the same region (AS-PCR, cited in 17 out of 56 papers since 2006, see Additional file 2) or the restriction of form-specific SNPs in the IGS rDNA region (IGS^581 ^and IGS^690 ^PCR-RFLPs: cited in [24] and [2], respectively, Additional file 2). In most of these papers only a single identification approach was used, while in four of them both AS-PCR and PCR-RFLP^581 ^methods were applied. Moreover, a few authors (nine papers; Additional file 2) still exploited the original approach developed by Favia *et al *[15], which is based on the same restriction site of Fanello *et al *[16], but requires the pre-identification of *A. gambiae *s.s. specimens. Overall, so far, the general attitude has been to consider all these approaches fully interchangeable; however, recent data from the westernmost extreme of M and S range (i.e. The Gambia and Guinea Bissau [21]) have highlighted that this assumption is not correct. The results here presented confirm this conclusion and allow to pinpoint the bases of the observed inconsistencies among results of the three approaches, as follows.

### Incorrect match of M and S specific primers used in the AS-PCR approach

A higher number of MS heterozygous patterns resulted from AS-PCR than from PCR-RFLP genotyping, mostly in specimens from Burkina Faso, Guinea Bissau and The Gambia. This is due to a low specificity of the AS-PCR approach (using form-specific primers differing only for the SNP variant at 3' end) which is affected by the inability of this single 3' mismatch to prevent extension of the non-specific primer by the polymerase [23]. In fact, this low specificity has been recently circumvented using primers containing an additional intentional mismatch at the third nucleotide from the 3' end which increases the power of *Taq *polymerase to extend from the 'right' primer and to partly optimize the reaction thermodynamics when both primers anneal on the template, thus providing more power to identify MS hybrids (IMP-PCR [14]). The comparison between the results of the AS-PCR and of IMP-PCR highlighted a higher specificity of the latter. In fact, IMP-PCR produced patterns consistent with those obtained either by sequencing or by the two PCR-RFLP approaches in all specimens tested (N = 146), with the exception of two S-form specimens from Gambia genotyped as MS by IMP-PCR (Table 2, line 18).

### Presence of polymorphisms in the recognition sequence of restriction enzymes used in the PCR-RFLP approaches

An A/C heterozygous pattern was observed in the recognition sequence of the enzyme utilised in IGS^690 ^PCR-RFLP (i.e. *MseI*) in five out of 32 M-form specimens from Angola. This polymorphism did not allow the complete cleavage of the M-specific PCR-amplified band, thus producing a false heterozygous MS^690 ^pattern.

### Incomplete cleavage during the restriction reaction

A few specimens from Burkina Faso, Cameroon, Guinea Bissau and The Gambia were incorrectly genotyped as MS by PCR-RFLP (IGS^581^: N = 8; IGS^690^: N = 5), due to incomplete digestion of the PCR-amplified fragment during restriction. A second round of PCR-RFLP reactions did not change the observed PCR-pattern and the specimens were confirmed to be homozygous at each site by sequencing.

### Presence of different number of copies of M and S-specific IGS-arrays in single individuals

This has been already hypothesized by Caputo *et al *[21] based on the inconsistent results from PCR-RFLP^581 ^and SINE-PCR on samples from Guinea Bissau and The Gambia, where a secondary contact zone between the two molecular forms has been hypothesized based on the high frequencies of MS putative hybrids reported [19,20]. The results obtained confirm this hypothesis and highlight the technical bias which emerged when the same samples were identified by PCR-RFLP^690^. In fact, the restriction enzyme used for the PCR-RFLP^581 ^(i.e. *HhaI*) recognizes a S-specific restriction site, while the enzyme used for the PCR-RFLP^690 ^(i.e *MseI*) recognizes a M-specific restriction site. It is possible to hypothesize that the PCR-amplification of individuals characterized by a number of copies of the M-IGS type higher than of S-IGS type exponentially increases this difference, producing a strong M^581 ^band and a weak S^581 ^one. The latter may not be visible on the agarose gel after the restriction step resulting in a MM^581^/MS^690 ^RFLP pattern. Conversely, individuals characterized by a number of copies of the S-IGS type higher than of M-IGS type are likely to produce a MS^581^/SS^690 ^RFLP pattern. This hypothesis is further supported by the relative high frequency of MM^581^/MS^690 ^(9%) and MS^581^/SS^690 ^(12%) specimens in the sample analysed, and by the absence of SS^581^/MS^690 ^and MS^581^/MM^690 ^genotypes. The QSV analysis of IGS sequences confirms that MM^581^/MS^690 ^or MS^581^/SS^690 ^individuals have proportions of array copy number intermediate between those of either MM^581^/MM^690 ^and MS^581^/MS^690 ^or SS^581^/SS^690 ^and MS^581^/MS^690 ^individuals, respectively (Table 2).

The comparison between the results of the IGS-genotyping (including direct sequencing, in case of inconsistencies among the approaches utilized) and of SINE-PCR showed consistent identifications in all samples, with the exception of those from Guinea Bissau and The Gambia. In these populations mismatches were observed, mostly due to SS and MM SINE-homozygotes with a heterozygous MS^IGS ^genotype or, less frequently, to MS SINE-heterozygotes with MM^IGS ^and SS^IGS ^genotypes, while no opposite MM-SINE/SS^IGS ^or SS-SINE/MM^IGS ^were found. As discussed in Caputo *et al *[21], the former individuals are likely to represent Fn progenies of inter-form crosses occurring in this "secondary contact zone", where the reproductive isolation mechanisms between M- and S-forms appear to be less effective than in the rest of the molecular forms sympatric distribution range. In fact, discrepancies between results from PCR-RFLP^581 ^and SINE-PCR led to hypothesize that the high frequencies of MS^581 ^patterns found in Guinea Bissau and in The Gambia were due to the presence of both M- and S-arrays in the multi-copy IGS rDNA region of single individuals, suggesting inter-locus recombination [21]. In this scenario, the SINE-PCR genotyping allows to discriminate putative MS hybrids from progenies of Fn-backcrosses (i.e. MM or SS SINE-homozygotes showing both M- and S-specific IGS arrays). In fact, the SINE-PCR genotyping of four MS^581 ^specimens reported in della Torre *et al *[7] (from Benin, Mali, Guinea and The Gambia) confirmed their putative hybrid origins.

On the other hand, the finding of high frequencies of consistent MS IGS/SINE patterns in larval samples from Burkina Faso, led Riehle *et al. *[24] to carry out a deeper genetic characterization of these individuals and to hypothesize that they may represent a new *A. gambiae *"sub-form" highly differentiated from M and S. This "sub-form" seems to be also characterized by a MS SINE-polymorphism in Hardy-Weinberg equilibrium consistent with IGS-patterns, a very unexpected scenario which needs to be taken into consideration when speculating on the origin of this putative "sub-form". In fact, based on their evolutionary dynamics, both IGS and SINE markers are expected to undergo rapid fixation in a randomly mated diverging taxa rather than being found at equilibrium in a taxon separated from M- and S-form.

Overall, the results here presented, as well as those by Riehle *et al *[24], do not only highlight limits in the approaches currently applied to discriminate M- and S-forms, but also on the actual definition of the two molecular forms, which might not fully correspond to the two *A. gambiae *incipient species in their entire geographical range. The M and S molecular forms are, in fact, defined specifically based on SNPs in the IGS region, which were initially used to discriminate between Mopti and Savanna/Bamako chromosomal forms in Mali and Burkina Faso [15] and, later, to identify two incipient species in other geographical regions, where the correlation with specific karyotypes was more complex [1,5,7,25]. Since their initial description, all data on the genetic, ecological and behavioural divergence of M and S forms were obtained based on the IGS diagnostics, leading to a general acceptance of the IGS-SNPs as form-specific characters possibly linked to genes or genomic regions instrumental to the *speciation process*. This view was reinforced by the fact that the IGS lies within X-chromosome centromeric region, where most genetic divergence between M- and S-forms is observed [11,12,25,26] and by the consistent almost complete absence of MS^IGS ^genotypes in nature. The finding of different number of copies of M- and S- IGS-arrays in single individuals from the western extreme of the molecular form range [21] highlighted how the genetic definition of the two *A. gambiae *incipient species is not fully tenable along their entire range. The recent sequencing of the genome of M- and S-colonies from Mali [11] and the availability of affordable SNP micro-array platforms [12], will probably allow in the near future a relatively easy processing of *A. gambiae *populations from the entire range. Moreover, the likely detection of multiple markers along their M-and S-form genome and their association will possibly allow a more precise definition of the two incipient species, as in the case of the allelic variant of TEP1 immune gene found to be fixed in M samples from Mali and Burkina Faso but absent in sympatric S populations [27].

## Conclusion

The results obtained reveal that the PCR and PCR-RFLP approaches most commonly utilized to identify *A. gambiae *molecular forms are not fully interchangeable, as usually assumed. Different kinds of technical biases have been highlighted, which may result in an overestimation of MS putative hybrids. This is particularly relevant in settings of realised gene flow between molecular forms, such as the areas at the extreme West African distribution of *A. gambiae*. Moreover, the IMP-PCR developed by Wilkins *et al *[14], and so far applied almost exclusively on laboratory samples, was shown to be more specific than AS-PCR thus encouraging its exploitation in large scale screenings of field *A. gambiae *samples. However, the risk of biases due to local polymorphisms in the annealing sequences should be always be taken into consideration.

From an operational perspective, it needs to be highlighted that the choice of the most convenient method for large-scale M- and S-form identification, also depends from technical considerations (e.g. laboriousness of the different approaches) and from the sympatric presence of other members of the *A. gambiae *complex in the study area. In fact, only IGS^581 ^PCR-RFLP allows the simultaneous identification of all species and molecular forms and could be the method of choice whenever the presence of other *A. gambiae *complex members (i.e. *Anopheles melas, Anopheles merus, Anopheles quadriannulatus *and/or *Anopheles bwambae*) cannot be excluded. Alternatively, the IMP-PCR approach could be used after *A. gambiae *s.s. specimens are identified by the species-specific PCR developed by Scott *et al *[28], thus avoiding the risks connected to the restriction step in IGS^581 ^PCR-RFLP. The choice of one or the other approach should also be linked to the relative frequencies of *A. gambiae *s.s. in the sample (i.e. if this frequency is low, the species-specific PCR + IMP-PCR approach could be more convenient, as only few specimens would require to be identified by IMP-PCR; if high, the IGS^581 ^PCR-RFLP could be a more straightforward approach). It should be noted that the IGS^690 ^PCR-RFLP is more sensitive in cases of degraded DNA samples and that it could simultaneously identify M- and S-forms and *A. arabiensis *[17]. On the other hand, the use of AS-PCR would require preliminary identification of *A. gambiae *s.s. specimens and is shown to be subject to more biases than the other approaches. The PCR-RFLP originally developed by Favia *et al *[15] and still recently utilized by some authors (see Additional file 2) is comparatively less suitable for large-scale studies as it requires previous complex species identification and yet it targets the same SNP as the PCR-RFLP^581^. The latter method is more efficient since it allows for simultaneous species and molecular form identification on a much smaller amplicon (367 bp compared to 1.3 kb [15]).

It is also proposed that, due to the straightforward amplification of small DNA fragments (i.e. 249 and 479 bp for S- and M-forms, respectively), SINE-PCR could be conveniently applied to easily identify M- and S-forms (even without preliminary species-specific PCR identification in areas where exclusive sympatry with *A. arabiensis *is found). However, it is important to keep in mind that the M-form specific SINE insertion is a character linked to the IGS-SNPs defining the M- and S-forms along most of their range, but with a different evolutionary history (i.e. its origin and rapid fixation in M-form).

Finally, it is recommend to apply more than one genotyping approach (and/or sequencing of the IGS-amplicon) when identifying samples from previously unexplored geographic areas within M- and S-form sympatric range and whenever MS hybrid patterns are observed (Additional file 3). In this latter case, in fact, presence of both M- and S-specific IGS arrays in single individuals could lead to a misleading calculation of frequency of hybridization between M and S forms, as shown in populations from Guinea Bissau and The Gambia, where the simultaneous use of SINE-PCR allowed a better understanding of the local situation.

## Abbreviations

**IGS**^**581 **^**SNP**: T/C Single Nucleotide Polymorphism at position 581 of IGS rDNA region; **IGS**^**690 **^**SNP**: A/T Single Nucleotide Polymorphism at position 690 of IGS rDNA region; **PCR-RFLP**^**581**^: PCR-RFLP recognising IGS^581 ^SNP; **PCR-RFLP**^**690**^: PCR-RFLP recognising IGS^690 ^SNP; **AS-PCR**: PCR based on Allele-Specific primers designed on IGS^581 ^SNP; **SINE-PCR**: PCR approach based on the specific and irreversible single-locus insertion of a Short Interspersed Transposable Element in the X-chromosome centromeric region; **IMP-PCR**: PCR based on Intentional Mismatch Primers annealing on IGS^581 ^and IGS^690 ^SNPs; **CNP**^**581**^: Copy Number Proportion of T/C alleles at positions 581 in the IGS amplicon; **CNP**^**690**^: Copy Number Proportion of A/T alleles at positions 690 in the IGS amplicon; **MM**^**581**^, **SS**^**581**^, **MS**^**581**^: M- and S-form specific genotypes at IGS^581 ^SNP; **MM**^**690**^, **SS**^**690**^, **MS**^**690**^: M- and S-form specific genotypes at IGS^690 ^SNP; **MM-SINE**, **SS-SINE**, **MS-SINE**: M- and S-form specific SINE genotypes.

## Competing interests

The authors declare that they have no competing interests.

## Authors' contributions

FS, MC and JLV carried out the molecular genotyping and participated in the sequence alignment. FS, BC and EM performed the statistical analyses. VP and JP participated in the design of the study and contributed to data analyses. AdT conceived of the study, participated in its design and coordination, and drafted the manuscript. FS, BC, MC, EM, VP and JP contributed to the manuscript finalization. All authors read and approved the final manuscript.

## Supplementary Material

Additional file 1 **P values of pairwise comparisons of CNP**^**581 **^**and CNP**^**690 **^**scores **The data show P values of pairwise comparisons of CNP^581 ^and CNP^690 ^scores calculated by QSV analyser in *Anopheles gambiae *specimens classified by IGS^581^/IGS^690 ^PCR-RFLPs. Click here for file

Additional file 2 **Papers on *Anopheles gambiae *M and S molecular forms published since 2006 **[2-4,9,13,15-17,19,20,24,25,32-70]. Papers focused on *Anopheles gambiae *M and S molecular forms are listed to highlight the identification methods mostly utilized in the last five years. Click here for file

Additional file 3 **Frequency of M and S molecular forms and putative MS hybrids in 10/56 papers on *Anopheles gambiae *s.s. published since 2006**. Click here for file
