# Supplementary material for: Comparative analyses reveal discrepancies among results of commonly used methods for Anopheles gambiaemolecular form identification
Source: Malar J. 2011 Aug 2;10:215. doi: 10.1186/1475-2875-10-215 (PMC3170251; doi:10.1186/1475-2875-10-215)
Supplement: Additional file 1 — P values of pairwise comparisons of CNP581 and CNP690 scores The data show P values of pairwise comparisons of CNP581 and CNP690 scores calculated by QSV analyser in Anopheles gambiae specimens classified by IGS581/IGS690 PCR-RFLPs. [file 1475-2875-10-215-S1.DOC]

**Additional File 1** - **P values of pairwise comparisons of CNP^581^ (a) and CNP^690^ (b) scores** calculated by QSV analyser [51] in *Anopheles gambiae* specimens classified by IGS^581^/IGS^690^ PCR-RFLPs: the underlined SS/SS (N=9) and MM/MM (N=15) groups correspond to S-form and M-form specimens from Burkina Faso and Angola, while the not-underlined groups correspond to specimens from The Gambia and Guinea Bissau (SS-SS: N=15; MS-SS: N=14; MS-MS: N=36; MM-MS: N=11; MM-MM: N=15). Statistically significant P values are in bold.

a)

| IGS CNP ^581^ | SS/SS | SS/SS | MS/SS | MS/MS | MM/MS | MM/MM | MM/MM |
| --- | --- | --- | --- | --- | --- | --- | --- |
|
| SS/SS |  |  |  |  |  |  |  |
| SS/SS | 1.000 |  |  |  |  |  |  |
| MS/SS | 0.384 | 0.730 |  |  |  |  |  |
| MS/MS | **0.002** | **0.000** | 1.000 |  |  |  |  |
| MM/MS | **0.000** | **0.000** | 0.122 | 1.000 |  |  |  |
| MM/MM | **0.000** | **0.000** | **0.000** | **0.000** | 1.000 |  |  |
| MM/MM | **0.000** | **0.000** | **0.000** | **0.001** | 1.000 | 1.000 |  |

b)

| IGS CNP ^690^ | SS/SS | SS/SS | MS/SS | MS/MS | MM/MS | MM/MM | MM/MM |
| --- | --- | --- | --- | --- | --- | --- | --- |
|
| SS/SS |  |  |  |  |  |  |  |
| SS/SS | 1.000 |  |  |  |  |  |  |
| MS/SS | 1.000 | 1.000 |  |  |  |  |  |
| MS/MS | **0.015** | **0.001** | 1.000 |  |  |  |  |
| MM/MS | **0.000** | **0.000** | **0.010** | 0.369 |  |  |  |
| MM/MM | **0.000** | **0.000** | **0.000** | **0.000** | 1.000 |  |  |
| MM/MM | **0.000** | **0.000** | **0.000** | **0.001** | 1.000 | 1.000 |  |
