# Supplementary material for: Comparative analyses reveal discrepancies among results of commonly used methods for Anopheles gambiaemolecular form identification
Source: Malar J. 2011 Aug 2;10:215. doi: 10.1186/1475-2875-10-215 (PMC3170251; doi:10.1186/1475-2875-10-215)
Supplement: Additional file 2 — Papers on Anopheles gambiae M and S molecular forms published since 2006 [2-4,9,13,15-17,19,20,24,25,32-70]. Papers focused on Anopheles gambiae M and S molecular forms are listed to highlight the identification methods mostly utilized in the last five years. [file 1475-2875-10-215-S2.DOC]

**Additional file 2 - Papers on *Anopheles gambiae* M and S molecular forms published since 2006.**

In 56 papers focused on *Anopheles gambiae* M and S molecular forms published since 2006, one among the following three identification approaches was used: PCR-RFLP^581^ [16] was used 24 times [3, 19, 20, 24, 45-64]; AS-PCR [13] 17 times [2, 9, 25, 38, 65-77] and PCR-RFLP^690^ [17] 2 times [4, 79]. Four papers utilised both PCR-RFLP^581^ and AS-PCR approaches [41-44]. Unexpectedly, the PCR-RFLP original approach developed by Favia *et al.* [13] was applied in 9 papers [32-40], despite being outdated, as based on the same restriction site of PCR-RFLP^581^, but requiring a pre-identification of *A. gambiae* s.s. specimens. Additional file 2 reports the only 10 papers, out of the above 56 referenced ones, where putative MS individuals were found.
