# Supplementary material for: Comparative analyses reveal discrepancies among results of commonly used methods for Anopheles gambiaemolecular form identification
Source: Malar J. 2011 Aug 2;10:215. doi: 10.1186/1475-2875-10-215 (PMC3170251; doi:10.1186/1475-2875-10-215)
Supplement: Additional file 3 — Frequency of M and S molecular forms and putative MS hybrids in 10/56 papers on Anopheles gambiae s.s. published since 2006. [file 1475-2875-10-215-S3.DOC]

**Additional file 3 – Frequency of molecular forms and putative hybrids in 10/56 papers on *Anopheles gambiae* s.s. published since 2006.**

| References | Country | Method | M-form | S-form | Hybrids | TOT |
| --- | --- | --- | --- | --- | --- | --- |
| Caputo *et al* 2008 [19] | The Gambia | PCR-RFLP^581^ and PCR-RFLP^690^ | 69.1% | 29.3% | 1.6% | 2364 |
| Oliveira *et al* 2008 [20] | Guinea Bissau | PCR-RFLP^581^ | 38.4% | 40.1% | 21.5%* | 172 |
| Ndiath *et al* 2008 [69] | Senegal | AS-PCR | 42.5% | 54.5% | 3% | 743 |
| Vezenegho *et al* 2009 [39] | Guinea Conakry | Favia *et al* 1997 | 28% | 71.8% | 0.2% | 465 |
| Costantini *et al* 2009 [2] | Burkina Faso | AS-PCR | 36.6% | 62.4% | 0.94% | 3866 |
| Ranson *et al* 2009 [49] | Burkina Faso | PCR-RFLP^581^ | 19.6% | 79% | 1,4% | 145 |
| Djogbénou *et al* 2010 [36] | Benin | Favia *et al* 1997 | 26.5% | 73.3% | 0.2% | 1717 |
| Dery *et al* 2010 [35] | Ghana | Favia *et al* 1997 | 11.4% | 77.2% | 11.4% | 35 |
| Choi *et al* 2010 [66] | Republic of Congo | AS-PCR | 0 | 98% | 2%* | 52 |
| Riehle *et al* 2011 [24] | Burkina Faso | PCR-RFLP^581^ | 51% | 27.5% | 21.5% | 1017 |

*Hybrid confirmed by sequencing
